# A ribonucleoprotein transfection strategy for CRISPR/Cas9‐mediated gene editing and single cell cloning in rainbow trout cells

**DOI:** 10.1186/s13578-021-00618-0

**Published:** 2021-06-03

**Authors:** Marina Zoppo, Nicole Okoniewski, Stanislav Pantelyushin, Johannes vom Berg, Kristin Schirmer

**Affiliations:** 1 Eawag, Swiss Federal Institute of Aquatic Science and Technology, 8600 Dübendorf, Switzerland; 2 Institute of Laboratory Animal Science, University of Zurich, 8952 Schlieren, Switzerland; 3 ENAC, EPF Lausanne, 1015 Lausanne, Switzerland; 4 Department of Environmental Systems Science, ETH Zürich, 8092 Zürich, Switzerland

**Keywords:** Rainbow trout (*Oncorhynchus mykiss*), CRISPR/Cas9, Ribonucleoprotein (RNP) complex, RTgutGC, Cytochrome P450

## Abstract

**Background:**

The advent of the Clustered Regularly Interspaced Short Palindromic Repeats (CRISPR)/Cas9 technology marked the beginning of a new era in the field of molecular biology, allowing the efficient and precise creation of targeted mutations in the genome of every living cell. Since its discovery, different gene editing approaches based on the CRISPR/Cas9 technology have been widely established in mammalian cell lines, while limited knowledge is available on genetic manipulation in fish cell lines. In this work, we developed a strategy to CRISPR/Cas9 gene edit rainbow trout (*Oncorhynchus mykiss*) cell lines and to generate single cell clone-derived knock-out cell lines, focusing on the phase I biotransformation enzyme encoding gene, *cyp1a1,* and on the intestinal cell line, RTgutGC, as example.

**Results:**

Ribonucleoprotein (RNP) complexes, consisting of the Cas9 protein and a fluorescently labeled crRNA/tracrRNA duplex targeting the *cyp1a1* gene, were delivered via electroporation. A T7 endonuclease I (T7EI) assay was performed on flow cytometry enriched transfected cells in order to detect CRISPR-mediated targeted mutations in the *cyp1a1* locus, revealing an overall gene editing efficiency of 39%. Sanger sequencing coupled with bioinformatic analysis led to the detection of multiple insertions and deletions of variable lengths in the *cyp1a1* region directed by CRISPR/Cas9 machinery. Clonal isolation based on the use of cloning cylinders was applied, allowing to overcome the genetic heterogeneity created by the CRISPR/Cas9 gene editing. Using this method, two monoclonal CRISPR edited rainbow trout cell lines were established for the first time. Sequencing analysis of the mutant clones confirmed the disruption of the *cyp1a1* gene open reading frame through the insertion of 101 or 1 base pair, respectively.

**Conclusions:**

The designed RNP-based CRISPR/Cas9 approach, starting from overcoming limitations of transfection to achieving a clonal cell line, sets the stage for exploiting permanent gene editing in rainbow trout, and potentially other fish cells, for unprecedented exploration of gene function.

**Supplementary Information:**

The online version contains supplementary material available at 10.1186/s13578-021-00618-0.

## Background

The discovery of the Clustered Regularly Interspaced Short Palindromic Repeats (CRISPR)/Cas as part of an adaptive immune system in Bacteria and Archaea [1–3], and their subsequent adaptation for gene editing in human cells and other eukaryotes, has revolutionized the field of molecular biology [4–6]. In comparison to other gene editing techniques, such as zinc finger nucleases (ZFNs) and transcription activator-like effector nucleases (TALENS) [7], which necessitate the design and generation of new proteinaceous DNA binding domains for every new genomic target, the type II CRISPR/Cas9 system requires as little as two components: the *Streptococcus pyogenes* CRISPR-associated protein 9 (Cas9) and a guide RNA (gRNA). This simplicity makes the CRISPR/Cas9 system a more convenient, cost-effective and flexible tool for gene editing. The gRNA comprises 96 nucleotides, of which twenty at the 5′ end direct Cas9 to a complementary sequence of the target genomic locus via Watson–Crick base pairing. The perfect complementarity and the presence of a protospacer adjacent motif (PAM) site immediately downstream of the target sequence (5′-NGG-3′), allows Cas9 to induce a double strand break (DSB) three nucleotides before the PAM sequence. The DSB acts as a trigger for the activation of endogenous repair systems, which in eukaryotes can lead to the activation of two main mechanisms: the non-homologous end joining (NHEJ) and homology directed (HDR) repair pathways. In NHEJ, DNA strands are re-ligated in such a way that they include nucleotide insertions or deletions (indels), leading to frameshift mutations that commonly result in the premature termination of the encoded protein [8]. On the contrary, HDR uses a homologous repair template in order to introduce precise insertions in the gene of interest [9]. Despite its versatility, HDR has proven to be less efficient compared to the NHEJ pathway. For example, it may be necessary to temporarily or permanently inhibit key enzymes of the NHEJ pathway in order to promote the correct incorporation of the repair template at the DSB [10].

In fish aquaculture species, numerous examples of in vivo CRISPR/Cas9 gene editing applications exist, targeting traits such as growth, sterility and disease resistance. Such applications of CRISPR/Cas9 were reported in *Oncorhynchus mykiss* (rainbow trout) [11]*, Salmo salar* (Atlantic salmon) [12–15], *Ictalurus punctatus* (channel catfish) [16–18], *Labeo rohita* (rohu carp) [19] and *Cyprinus carpio* (common carp) [20] (reviewed in [21]). In these instances, the delivery of the CRISPR/Cas9 components was accomplished through microinjection of the mRNA encoding for Cas9 and a gRNA targeting the gene of interest into the fertilized egg.

In contrast, CRISPR/Cas9 editing in vitro, using fish cell cultures, is much less explored. The overall workflow for CRISPR genome editing includes several steps, such as the design of the gRNA for targeting the gene of interest, cell transfection, determination of the gene editing efficiency, establishment of single-cell clones, and, finally, characterization of the mutant cell line(s). For each of these steps, there are hurdles to overcome, especially for fish cell lines, which are notorious for their low transfection efficiencies [22, 23]. The cell membrane, which constitutes one of the main barriers to gene delivery, shows unique feature in fish cells. In fact, the lower temperatures of incubation and the increased saturation of the phospholipids in the cellular membrane compared to mammalian cells, make the fish phospholipid bilayer more rigid and therefore, highly refractory to DNA entry [24].

The first evidence of CRISPR/Cas9 gene editing in a Chinook salmon (*Oncorhynchus tshawytscha*) cell line was presented in 2016 [25]. In this study, the embryonic cell line, CHSE-EC, which was derived from CHSE-214, was stably transfected to express both the enhanced green fluorescent protein (EGFP) and Cas9. Expression of a gRNA targeting the EGFP gene resulted in a gene targeting efficiency of 34.6%. The above-mentioned approach was employed later by the same group in a follow-up study aimed at dissecting the type I and II interferon signaling through *stat2* gene inactivation [26]. A similar gene editing strategy was adopted more recently by Escobar-Aguirre and colleagues with the development of an all-in-one vector for the transient expression of the CRISPR/Cas9 elements, again in the CHSE-214 cell line [27]. As mentioned by the authors, only 10% of transfected CHSE-214 cells received the CRISPR vector, due to the intrinsic lower transfection efficiency of fish cells and most probably also because of the large size of the plasmid (14 Kb). Several reports on a plasmid-based CRISPR/Cas9 system are available in *Cyprinidae* fish cell lines, focusing on the potential application of gene editing techniques against common aquaculture viral infections, such as the cyprinid herpesvirus-3 (CyHV-3) in the common carp caudal fin (KF-1) cell line [28] and the grass carp reovirus (GCRV) in the grass carp (*Ctenopharyngodon idella*) kidney (CIK) cell line [29]. Similarly, the CRISPR/Cas9 technology can be employed for cost-effective production of fish vaccines. A first step towards this goal was achieved by Kim and colleagues with the generation of a gene edited Epithelioma Papulosum Cyprini (EPC) cell line able to produce high titers of viral hemorrhagic septicemia virus (VHSV) [30].

A valid alternative to plasmid delivery is represented by viruses whose natural ability to deliver nucleic acids inside the cells can be harnessed to introduce the gRNA and/or Cas9 into the cells. Adeno-associated virus (AAV), adenovirus (AV) and lentivirus (LV) are commonly used as CRISPR/Cas9 delivery tools for mammalian cell lines and for in vivo gene editing [31]. To date, the only example of CRISPR/Cas9 delivery using viral vectors in fish cell lines is represented by the work of Gratacap and colleagues, where a lentivirus-based method for CRISPR/Cas9 mediated gene editing in the CHSE-214 cell line was optimized, achieving a gene editing efficiency of 90% [32].

However, despite the high transfection efficiency, several concerns related to the usage of viral vectors have been raised in the last decade. Prolonged expression of the CRISPR/Cas9 elements leads to an increased probability of off-target effects and insertional mutagenesis events due to the integration of the viral DNA into the host genome are main drawbacks of this strategy [33]. By contrast, transfection of a ribonucleoprotein (RNP) complex consisting of the Cas9 protein combined with the desired gRNA avoids most of the hurdles encountered with plasmid or viral delivery. The direct delivery of RNPs, on the one side, accelerates the gene editing kinetics [34] and, on the other side, mitigates the off-target effects due to the rapid degradation of Cas9 [34–36]. Moreover, owing to the DNA-free nature of the RNP approach, potential transgene insertions are avoided. RNP transfection has been successfully applied for the first time in medaka (*Oryzias latipes*) fish cells with a reported mutation efficiency of 61.5% [37]. Moreover, a CRISPR/Cas9 approach based on RNP transfection has recently been developed for different salmonid cell lines of Atlantic salmon (SHK-1 and ASK), Rainbow trout (RTG-2) and Chinook salmon (CHSE-214) with gene editing efficiencies of over 90% [22]. Due to the slow growth nature of salmonid cells and intrinsic difficulties in isolating clonal fish cell lines [23], clonal expansion of mutated SHK-1 cells was unsuccessful, and single cell cloning of the remaining cell lines used in the study, rainbow trout included, was not performed [22]. However, for later phenotypic analysis, the establishment of a clonal cell population is a prerequisite, especially when dealing with low gene editing efficiencies.

Several methods are available for the generation of monoclonal cell lines. One cost-effective yet labor-intensive technique, which has been used for decades for the production of hybridomas [38], is represented by the limiting dilution method. This method relies on the dilution of the heterogeneous population and the statistical probability of depositing a single cell per well. An alternative and commonly used method to achieve single cell cloning is by using fluorescence-activated cell sorting (FACS) [39] or, less frequently, more sophisticated technologies such as Laser Capture Microdissection (LCM) [40], microfluidic devices [41, 42] and Magnetic-Activated Cell Sorting (MACS) [43]. Before the advent of automated single cell isolation methods, the use of cloning cylinders, together with the limiting dilution method, represented one of the most important approaches for the production of clonal cell lines [44–47].This approach has been recently used for the establishment of human multipotent progenitor cells [48] and the isolation CRISPR edited mammalian cell lines [49]. However, single clone isolation by means of cloning cylinders in fish cells has not yet been reported.

FACS and limiting dilution methods have been successfully used to generate single cell clones following CRISPR-mediated gene editing experiments in carp [30], grass carp [29], medaka [37] and chinook salmon [26] fish cell lines. When clonal isolation was not performed, gene editing was validated by amplification of the gene of interest by polymerase chain reaction (PCR), insertion into a cloning vector and Sanger sequencing of the resulting recombinant plasmids [25, 28, 37]. Clonal isolation of gene edited rainbow trout cells has not been reported so far, with the exception of luciferase gene transfected clones of the rainbow trout gonad cell line RTG-2 by the limiting dilution method [50].

The intestine-derived rainbow trout cell line RTgutGC [51] represents the only thus far characterized intestinal epithelial cell line from fish, and as a result, is widely used in both toxicological [52, 53] and aquaculture research studies [54, 55]. In the present work, a genetically engineered in vitro model based on the RTgutGC cell line, was developed using the CRISPR/Cas9 technology. CRISPR/Cas9 editing by electroporation of RNP complexes allowed generating homogeneous and long-term knock-out cell cultures that can be preserved and expanded for later use. The *cyp1a1* gene, encoding for the cytochrome P450, family 1, subfamily A, polypeptide 1, was chosen as first target for the generation of RTgutGC mutated cells. Finally, a method to isolate gene edited single cell clones of rainbow trout cells by means of cloning cylinders was developed for the first time.

## Results

### Electroporation of RTgutGC cells and enrichment of transfected cells

**Fig. 1:**
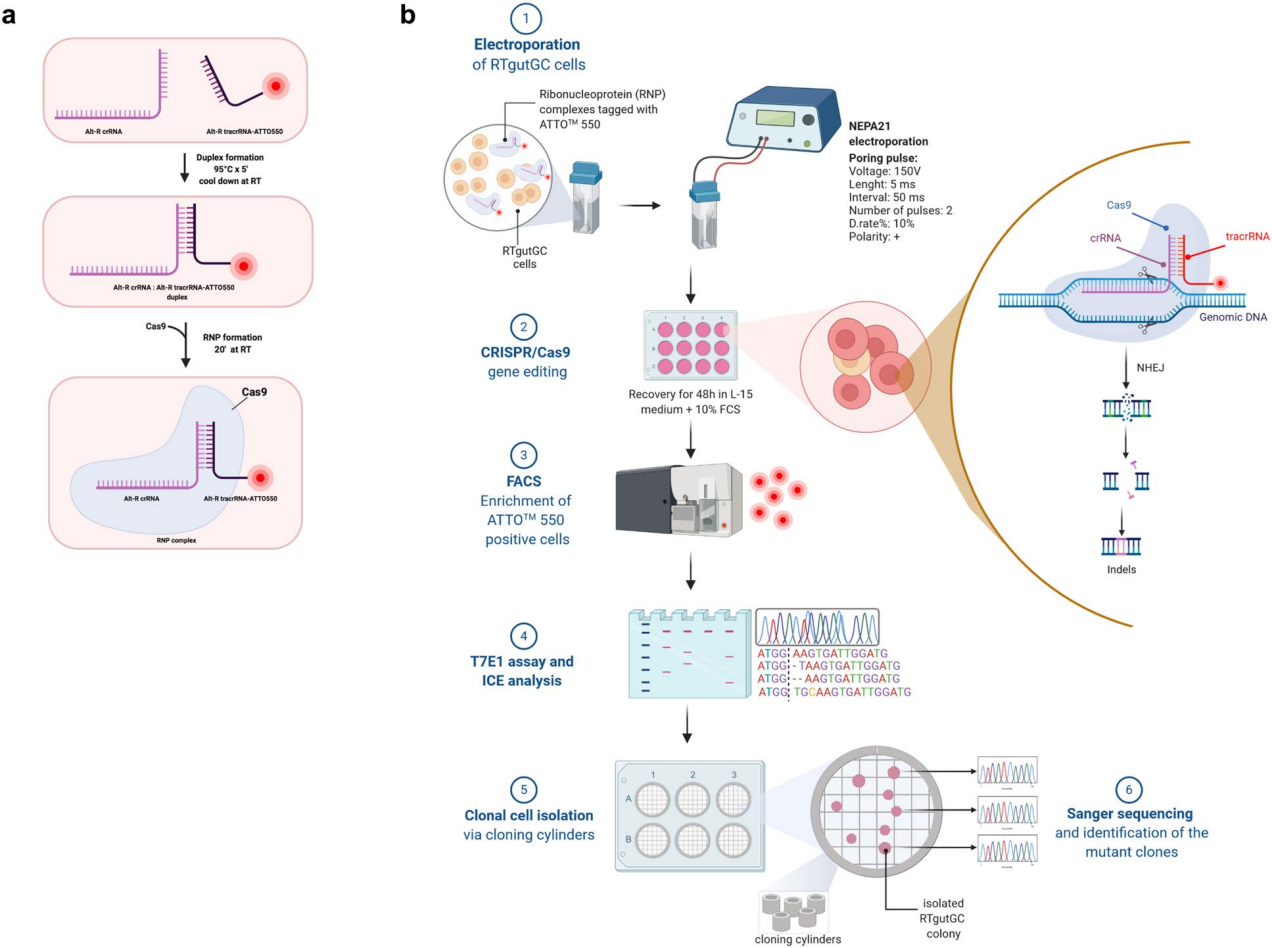
Ribonucleoprotein (RNP) complex transfection in RTgutGC cells. **a** Overview of the ribonucleoprotein (RNP) complex preparation. Alt-R®crRNA and Alt-R®tracrRNA-ATTO™ 550 are combined at equimolar concentration. Once the duplex crRNA and tracrRNA is formed, *Streptococcus pyogenes* Cas9 protein is added in order to form the RNP complex. **b** Overview of the CRISPR/Cas9 gene editing strategy workflow. Briefly, RNP complexes targeting rainbow trout *cyp1a1* were transfected in RTgutGC cells via electroporation using the NEPA21 electroporator. Following 48 h of recovery, transfected RTgutGC cells were sorted via FACS using the ATTO™ 550 signal and incubated in a 96 well plate. T7 endonuclease assay and ICE analysis were performed in order to evaluate the overall gene editing efficiency and the nature of the CRISPR/Cas9-induced mutations, respectively. Finally, clonal cell isolation was obtained through low cell density seeding and colony isolation using cloning cylinders. Created with https://biorender.com

CRISPR/Cas9 gene editing of the *cyp1a1* gene was accomplished in rainbow trout RTgutGC cells via electroporation with RNP complexes constituted by the Cas9 protein and crRNA:tracrRNA-ATTO™550 duplex. Figure 1a shows a schematic representation of the RNP preparation. Among all the candidates generated by the web tool CHOPCHOP [56], as listed in Additional file 1: Table S1, the crRNA selected for this study had the highest efficiency score among the sequences located in the first exon of the *cyp1a1* gene and the lowest self-complementarity score. The analysis of the selected crRNA indicated a putative off-target site in *cyp1a3*, a gene belonging to the CYP1A subfamily and sharing 96% nucleotide identity with *cyp1a1*. Nevertheless, the presence of a single base pair mismatch in the PAM-proximal region suggested that the off-target cleavage might be abolished. Following 48 h of recovery post electroporation, enrichment of transfected cells was performed through FACS, sorting the ATTO™ 550 positive cells in a 96-well plate. Cells subjected to RNPs but without electroporation were used as background controls and were analyzed first. A high background of ATTO™ 550 positive cells was detected, suggesting an unspecific binding of either the RNPs or the fluorophore to the RTgutGC cell surface (Additional file 1: Fig. S1). The analysis of this control allowed to appropriately set the gates for the isolation of bona fide transfected cells. As a direct consequence, only 2.2% of strongly positive transfected cells were selected during FACS. Despite numerous attempts, clonal expansion of RTgutGC cells after FACS failed. According to a purposefully performed titration experiment (Additional file 1: Fig. S2), a minimum amount of 2000 transfected cells per well was required to be sorted in a flat bottom 96-well plate, to yield a confluent monolayer following three weeks of incubation. Transfected cells were passaged sequentially to increasingly bigger cell-culture well plates and the genomic DNA was extracted for genotyping. An overview of the gene editing approach is depicted in Fig. 1b.

### Evaluation of the gene editing efficiency of *cyp1a1*

**Fig. 2:**
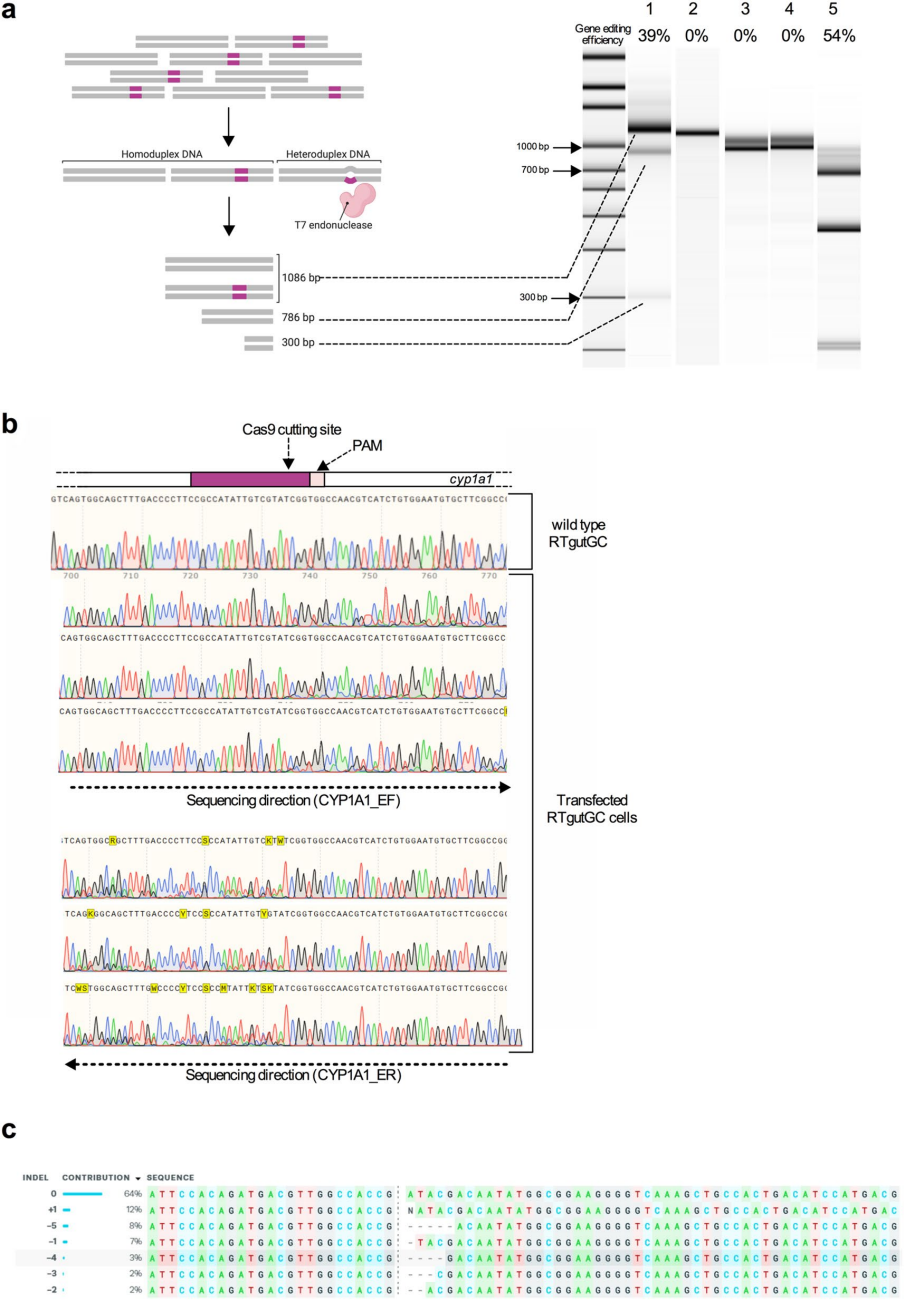
T7 endonuclease I (T7E1) assay and ICE analysis of RTgutGC transfected cells. **a** Left panel shows a schematic representation of the T7E1 assay. Right panel shows the results of the T7E1 assay visualized on an Agilent Bioanalyzer DNA High Sensitivity chip. 1: *cyp1a1* from RNP transfected RTgutGC cells; 2: *cyp1a1* from untransfected RTgutGC cells; 3: *cyp1a3* from transfected RTgutGC cells; 4: *cyp1a3* from untransfected RTgutGC cells; 5: T7E1 assay positive control. Uncut and cleaved fragments, corresponding to the *cyp1a1* wild type sequence and indels, respectively, are indicated with dashed lines. Gene editing efficiency is reported above each lane as percentage (%). **b** Sanger sequencing results of wild type (upper panel) and transfected (lower panel) RTgutGC cells. gRNA and PAM position are indicated by the purple and pink boxes, respectively. Cas9 cutting sites are indicated by vertical red lines*.* **c** Output of ICE analysis of the RNP transfected cells. The type of insertions and deletions (indels) detected in the analyzed sample and their respective frequencies are indicated on the left side. On the right side, the *cyp1a1* wild-type and edited sequences are aligned. Dashed line indicates the Cas9 cutting site. Created with https://biorender.com

To detect mutations induced by the CRISPR/Cas9 system in the *cyp1a1* gene, a T7E1 assay was performed. A schematic representation of the T7E1 assay workflow is illustrated in Fig. 2a. Genomic DNA of RNP transfected and untransfected RTgutGC cells was extracted and the *cyp1a1* locus amplified by PCR. If NHEJ repair events occurred following Cas9 DSB, denaturation and re-annealing of the PCR products will lead to the formation of wild type and heteroduplex PCR amplicons. T7 endonuclease I recognizes and cleaves non-perfectly matched DNA sequences contained in heteroduplexes. As shown in Fig. 2a, low molecular weight DNA bands of the correct size, corresponding to the heteroduplex digestion products, were detected when *cyp1a1* amplicons from RTgutGC transfected cells were analyzed. Evaluation of the intensity of the DNA bands revealed a gene editing efficiency of the *cyp1a1* locus of around 39%. As expected, *cyp1a1* amplicons derived from untransfected cells produced one DNA band corresponding to the uncut wild type product. No cleavage products were detected when the closely related gene, *cyp1a3,* was amplified from RNP transfected and untransfected RTgutGC cells. Overall, the T7E1 assay results clearly suggested that gene editing occurred in the *cyp1a1* locus when RNP complexes were transfected in RTgutGC cells. Moreover, the absence of digestion products of the *cyp1a3* amplicon confirmed the specificity of the crRNA selected for the RNP strategy.

### Sanger sequencing and Inference of CRISPR Edits

The *cyp1a1* DNA region targeted by the RNP complexes was amplified from transfected and untransfected RTgutGC cells and subjected to Sanger sequencing. Sequencing results further confirmed editing of the *cyp1a1* gene. More specifically, the analysis of the electropherograms produced from the sequencing of the RNP transfected cells indicated overlapping peaks in the region targeted by the crRNA. This finding suggests that multiple indels occurred at the Cas9 cutting site (Fig. 2b). On the contrary, sequencing of the untransfected cells yielded clean and evenly spaced peaks. In order to assess the nature and frequency of the different types of edits present in a population of mixed wild type and mutated cells compared to the wild type cells, the free bioinformatics tool Inference of CRISPR Edits (ICE) was used. As shown in Fig. 2c, analysis of the Sanger sequencing data obtained from RTgutGC transfected cells indicated the presence of indels of variable lengths (from − 5 nucleotides to + 1 nucleotides) detected at the Cas9 cutting site and therefore compatible with the NHEJ repair pathway. The overall percentage of editing detected by the ICE tool was 34%. An R^2^ value of 0.98 indicated a good correlation between the Sanger sequence data and the indel distribution proposed by the ICE tool.

### Clonal cell isolation of gene-edited RTgutGC cells via colony isolation

Despite numerous attempts, clonal cell isolation of RTgutGC cells via FACS or through a serial dilution method failed (data not shown). To overcome this obstacle and isolate pure CRISPR-edited clones of *cyp1a1*, we proceeded with low density seeding of ATTO™ 550 enriched cells (600–1200 cells/well) in 6-well plates followed by colony isolation using cloning cylinders.

**Fig. 3:**
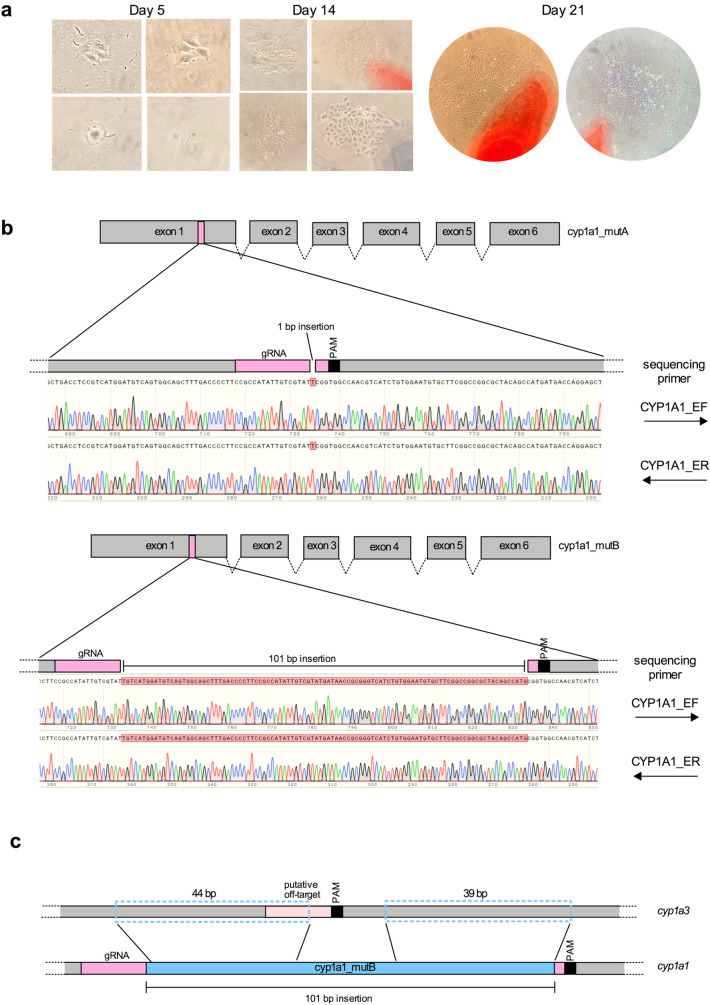
Isolation of RTgutGC clonal cell lines and sequencing results CRISPR/Cas9-gene edited cells. **a** Micrographs of RTgutGC colonies taken after 5, 14 and 21 days post low density seeding. **b** *cyp1a1* sequencing results of cyp1a1_mutA (upper panel) and cyp1a1_mutB (lower panel) gene-edited clones indicating 1 bp and 101 insertions, respectively. The gRNA position within *cyp1a1* gene sequence is schematically indicated below each sequencing result. **c** Schematic representation of the homology between the 101 bp insertion (blue rectangle) found in the targeted region of *cyp1a1* in the cyp1a1_mutB mutant cell line and the closely related gene *cyp1a3* (dashed blue rectangles)

Micrographs taken after 5, 14 and 21 days were acquired to detect single, isolated colonies (Fig. 3a). Once these colonies were large enough (300–400 cells), they were carefully isolated with the cloning rings and placed in wells of 96-well plates. Cells were allowed to recover in these 96-well plates for 3 weeks and were then propagated step-by-step into bigger well plates. Sanger sequencing of 15 randomly selected colonies led to the identification of two mutant clones, named cyp1a1_mutA and cyp1a1_mutB, respectively, bearing mutations in the *cyp1a1* locus. Sequencing results of the remaining colonies indicated 5 clones bearing the wild type *cyp1a1* gene and 8 colonies yielding mixed electropherogram signals in the *cyp1a1* region targeted by the RNP complexes. As shown in Fig. 3b, a 1 bp and 101 bp insertion, respectively, were identified three nucleotides before the PAM sequence. Both insertions in cyp1a1_mutA and cyp1a1_mutB mutants caused a frameshift of the *cyp1a1* open reading frame (ORF), resulting in the generation of several premature stop codons. The first premature stop codons were located 768 and 642 nucleotides after the ATG, in cyp1a1_mutA and cyp1a1_mutB, respectively. Interestingly, analysis of the 101 bp insertion in cyp1a1_mutB revealed two homology regions of respectively 44 bp and 39 bp, in the closely related *cyp1a3* gene (Fig. 3c). Sequencing of the only predicted off-target site in the *cyp1a3* gene did not show any differences between cyp1a1_mutA and cyp1a1_mutB mutants and wild type RTgutGC cells.

### Clonality assessment of *cyp1a1* gene-edited cells

**Fig. 4:**
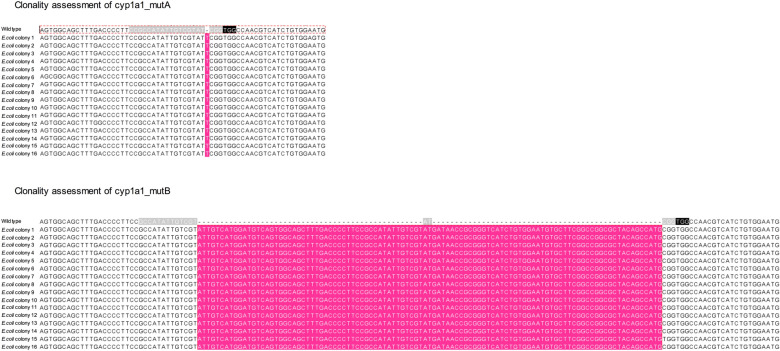
cyp1a1_mutA and cyp1a1_mutB clonality assessment. Upper and lower panels show the sequencing results obtained from 16 colonies following cloning of the *cyp1a1* gene from cyp1a1_mutA and cyp1a1_mutB, respectively, into pBluescript KS+. The 20 nucleotide sequences targeted by the crRNA is highlighted in grey, PAM sequence is highlighted in black, inserted sequences are highlighted in pink. All sequenced plasmids showed the expected genetic mutation in the *cyp1a1* region targeted by the crRNA

To confirm clonality of both cyp1a1_mutA and cyp1a1_mutB cell lines, the DNA region targeted by the crRNA was amplified using primers containing engineered SmaI restriction sites. The purified PCR products and pBluescript vector were digested with SmaI, ligated and transformed in competent *E. coli* cells. Screening of the ampicillin resistant colonies was performed via colony PCR and plasmid DNA (pDNA) extracted from the positive colonies. The sequencing results from 16 recombinant *E. coli* colonies, obtained for each cyp1a1_mutA and cyp1a1_mutB mutant cell line, indicated 1 bp and 101 bp insertion respectively, confirming the clonality of both the mutant cell lines and the robustness of the cloning cylinder isolation method (Fig. 4).

## Discussion

In this work we developed a CRISPR/Cas9 approach for gene editing of a rainbow trout cell line, RTgutGC, together with a method for clonal cell isolation of CRISPR edited rainbow trout cells. First, the most suitable parameters for the electroporation of RTgutGC cells were identified through an optimization experiment, in which voltage and duration of pulses were assessed. According to the results obtained, 150 V and 5 ms long pulses were the most suited parameters and therefore were selected for the transfection of RTgutGC cells. ATTO™ 550 labeled RNP complexes targeting a specific 20 nucleotide region located in the first exon of the *cyp1a1* gene were transfected via electroporation in the RTgutGC cell line. The choice of using a dual crRNA/tracrRNA format over the chimeric gRNA does not affect the gene editing performance, as already reported by studies aimed at comparing CRISPR/Cas9 efficiency using the two formats [57, 58]. FACS analysis of the background control, represented by untransfected RTgutGC cells incubated with fluorescently labeled RNP complexes, revealed a high percentage of cells displaying an ATTO™ 550 signal, indicating an unspecific interaction of either the RNP complexes or the dye with the surface of RTgutGC. This control allowed us to distinguish between untransfected and transfected cells. As reported by Liu and colleagues, electroporation of ATTO™ 550-labeled RNP complexes in medaka fish cell lines resulted in more than 90% transfected cells [37], however, the lack of a background control included in their experimental settings prevented the comparison with our results. The T7 endonuclease (T7E1) assay performed on the *cyp1a1* gene amplified from transfected cells indicated that gene editing occurred in the region of the DSB induced by Cas9. Analysis of the DNA band intensities revealed a gene editing efficiency of 39%. Due to the high degree of similarity between the selected crRNA and a region of the *cyp1a3* gene, the T7E1 assay was performed also on this gene. No additional DNA bands were observed when the *cyp1a3* gene was amplified from transfected cells, indicating that no off-target events occurred.

Comparison between the selected crRNA for *cyp1a1* mutagenesis and the potential off-target sequence in the *cyp1a3* gene indicated a single mismatch located six nucleotides before the PAM sequence. It is known that mismatches within the so-called “seed region” of the protospacer comprising 10 nucleotides proximal to the PAM site, are more likely to abolish target cleavage [59]. Therefore, it is tempting to hypothesize that the single mismatch in the *cyp1a3* region targeted by our crRNA was not tolerated by the CRISPR/Cas9 system and did not result in a Cas9 mediated cleavage in this gene.

Transfected RTgutGC cells were further investigated through Sanger sequencing of the *cyp1a1* locus. Mixed sequencing chromatograms were detected surrounding the DSB site, usually resulting from the superposition of different mutation profiles. In the absence of a donor template homologous to the sequence surrounding the cleavage, the DSB created by Cas9 is usually repaired using the NHEJ pathway, which seals the DNA break by randomly introducing indels [4, 60, 61]. Because of the unpredictability of the NHEJ pathway, and since this pathway repairs the DNA gap in a different way in each cell, the analysis of the sequencing results could not reveal any information regarding the genotype of the transfected cells. A quick approach to quantitatively assess the nature of the CRISPR edits when dealing with mixed cell populations utilizes gene editing analysis tools such as TIDE (Tracking of Indels by Decomposition) [62, 63] or ICE [64]. Using Sanger sequencing data, the ICE algorithm estimates the overall gene editing efficiency, as well as listing the profiles of the CRISPR edits and their relative abundance. Deconvolution analysis using the ICE tool indicated an overall gene editing efficiency of 34%, mostly constituted by 1 bp insertions. Nucleotide deletions ranging from -1 to -5 were also detected even though with lower frequency. Despite the fact that these values do not denote a high gene editing efficiency, in our experience the CRISPR/Cas9 gene editing approach developed in this study allows for higher gene editing efficiencies, close to 100%, when the target locus was changed (unpublished data). Further optimization of the crRNA can be achieved by combining different prediction programs or by performing in vitro gRNA validation [65]. In addition to the crRNA specificity, other factors, such as the target location in the genome and the epigenetic accessibility, can influence Cas9 cleavage performance. For example, nucleosomes, which constitute the basic structural units of the chromatin, are known to represent an impediment for Cas9 activity both in vitro and in vivo [66]. Moreover, CRISPR/Cas9 gene editing is significantly hampered if the target sequence is located in a heterochromatic region of the genome, highly packed and transcriptionally inactive [67]. It is possible that all of these factors, together with the selected crRNA, may have influenced the outcome of the *cyp1a1* gene targeting.

Because of the randomness of indels caused by the NHEJ pathway, different mutations in each cell can arise following the CRISPR/Cas9-mediated cleavage of the gene of interest. Moreover, indels can occur in-frame, resulting in a still partially functional protein. The coexistence of mutant and wild type cells expressing semi-functional or functional proteins, respectively, can represent an obstacle to the phenotypical characterization of the mutant cell lines, preventing the full understanding of the biological role of the protein. Finally, for any given target of interest it is not always possible to reach high gene editing efficiencies. For the above-mentioned reasons, it is imperative to derive clonal cell lines from the mixed transfected cell population.

Isolation of clonal RTgutGC cell lines via limiting dilution method or FACS failed despite numerous attempts. This is not the first example of failed clonal cell isolation in a fish cell line. In a recent study published by Gratacap and colleagues, describing CRISPR/Cas9 gene editing in multiple salmonid cell lines, single cell isolation of the head kidney Atlantic salmon cell line (SHK-1) was unsuccessful [22]. Several factors may be responsible for this behavior. These include the slow growth of the cell line and the need of growth factors released by other cells in culture, which is impossible to achieve when performing single cell cloning. A cost-effective method to avoid the latter obstacle is to seed the heterogeneous CRISPR-edited cell population at low density and isolate the colonies using cloning cylinders. Using this method, we achieved the isolation of two clonal RTgutGC cell lines bearing mutations in the *cyp1a1* gene. This is the first report of a clonal CRISPR edited cell line in rainbow trout and it sets the stage for future clonal isolation of gene edited rainbow trout and potentially other fish cell lines.

Compared to the parental heterogeneous population from which they originated, Sanger sequencing of the two CRISPR edited colonies, called cyp1a1_mutA and cyp1a1_mutB, yielded clean electropherogram peaks indicating 1 bp and 101 bp insertions, respectively. Interestingly, insertions of 1 bp were primarily recovered via ICE analysis in heterogeneous populations of rainbow trout gonad cell line (RTG-2) following electroporation of RNP complexes [22]. By contrast, repair of Cas9 induced DSB in cyp1a1_mutB led to a 101 bp sequence insertion with high degree homology with a similar region in *cyp1a3*. This repair pattern denotes the intervention of a repair mechanism different from the NHEJ and is compatible with the synthesis-dependent strand annealing (SDSA) pathway, a type of homologous recombination which occurs when one of the 3′ overhang of the DSB anneal and invade a complementary sequence which serves as template [68]. The SDSA pathway is also known for the ability of “capturing” and duplicating DNA sequences during the DSB repair [68]. The copied DNA can belong to extra chromosomal regions, such as plasmid or mitochondrial DNA [69], chromosome regions close to the breaking point [70] or, like in the case of *cyp1a3*, to regions located on another chromosome [70]. The sequence of *cyp1a3* identified as potential off target site during crRNA selection was sequenced in both cyp1a1_mutA and cyp1a1_mutB mutant cell lines. Sequencing results did not show any difference between the *cyp1a1* mutants and wild type RTgutGC cell line, further confirming the specificity of our RNP approach. Finally, in order to confirm the clonal isolation from a single progenitor cell, the *cyp1a1* gene was amplified from both CRISPR mutant cell lines and subcloned into the pBluescript SK+ plasmid. Transformation of the resulting vector into competent *E. coli* cells and sequencing of recombinant colonies confirmed the clonality of the obtained cell lines and the robustness of the colony isolation method.

## Conclusions

We developed a gene editing approach based on the CRISPR/Cas9 system in the intestinal rainbow trout (*Oncorhynchus mykiss*) cell line, RTgutGC. RNP complexes consisting of Cas9 and crRNA:tracrRNA duplex and targeting the *cyp1a1* gene were delivered via electroporation. Moreover, a method for clonal cell isolation based on cloning cylinders was used for the first time for rainbow trout cells. By applying this strategy, we successfully overcame the hurdles related with single cell cloning and identified two *cyp1a1* mutant clones. These clones have been preserved in liquid nitrogen from which they could be recovered for continued culture. Overall, the clonal *cyp1a1* mutant cell lines obtained in this study provide an excellent model to CRISPR/Cas9 gene edit and characterize the function of genes in rainbow trout. The establishment of a CRISPR/Cas9 platform for the precise editing of single genes also represents the first essential step required for the upgrade of a gene editing technology towards high-throughput studies, such as pooled and arrayed CRISPR screenings, which are currently revolutionizing diverse fields of biological sciences but have not yet been adapted in fish cell lines [71, 72].

## Material and methods

### Cell culture and media

The rainbow trout intestinal cell line, RTgutGC, originates from the distal intestine of a female rainbow trout (*Oncorhynchus mykiss*) [51]. Cells were routinely grown in 75 cm^2^ or 150 cm^2^ flasks (TPP, Trasadingen, Switzerland) in complete medium consisting of Leibovitz’s L-15 without phenol red (Invitrogen, Basel, Switzerland), supplemented with 5% fetal calf serum (FCS, Eurobio, Les Ulis, France) without the addition of antibiotics. The RTgutGC cells were cultured at 19 ± 1 °C in ambient atmosphere in the dark and sub-cultured every 7–10 days by washing the cells twice with Versene (Invitrogen, Basel, Switzerland) followed by trypsinization (Biowest, Nuaillé, France).

### Design of crisprRNA (crRNA) and preparation of ribonucleoprotein (RNP) complexes

The rainbow trout gene, *cyp1a1,* encoding for the Cytochrome P450, family 1, subfamily A, polypeptide 1, was selected as target for establishing the CRISPR/Cas9 gene editing strategy. A two-part gRNA version, comprised of a variable CRISPR RNA (crRNA), complementary at the 5’ end to the 20-nt-long genomic target sequence, in combination with a constant trans-activating CRISPR RNA molecule (tracrRNA), was utilized for *cyp1a1* targeting. crRNA design was performed using the CHOPCHOP online tool (https://chopchop.cbu.uib.no/), giving the priority to those targeting the first half of the coding sequence and with the highest on-target score. Other factors taken into considerations were the presence of potential off targets in the rainbow trout genome, the GC content, which should be between 40 and 70% [73, 74], and the self-complementarity of the selected crRNA sequence with the tracrRNA that might hinder the targeting of the gene of interest. The selected sequence for *cyp1a1* targeting (5′-CCGCCATATTGTCGTATCGG-3′) was purchased from IDT as crRNA in its proprietary Alt-R format (IDT, Coralville, IA, USA). ATTO™ 550-labeled transactivating RNAs (Alt-R®tracrRNA-ATTO™ 550, catalog number: 1072532) was likewise purchased from IDT. The dual crRNA/tracrRNA system was chosen over the single gRNA in order to benefit from a fluorescently-labeled tracrRNA which will allow, in the following steps, FACS based enrichment of the transfected cells. To generate the complete gRNA, Alt-R®crRNA and Alt-R®tracrRNA were reconstituted in nuclease-free duplex buffer (IDT) to a final concentration of 200 µM (Fig. 1a). Oligos were mixed at equimolar concentration (3 µl of Alt-R®crRNA and 3 µl of Alt-R®tracrRNA-ATTO™ 550) in a sterile PCR tube and incubated at 95 °C for 5 min followed by a cool down phase to room temperature (RT) for 20 min. In a PCR tube, 4 μl of crRNA:tracrRNA duplex were combined with 5 μl of Alt-R® *Streptococcus pyogenes* Cas9 Nuclease V3 (IDT, Cat. No. 1081058) and incubated at RT for 20 min to allow the formation of RNP complexes. Finally, 9 μl of RNP complexes (1.2 μM crRNA:tracrRNA, 1 μM Cas9 nuclease) were mixed with 6 μl of electroporation enhancer (IDT, Coralville, IA, USA) and added to the RTgutGC cells prepared as described below.

### RTgutGC transfection through electroporation

RTgutGC cells were transfected via electroporation using a NEPA21 electroporator (Sonidel limited, Ireland). The optimal electroporation conditions were identified through an optimization experiment in which RTgutGC cells were transfected with the plasmid pEGFP-C1-Flag (Addgene Plasmid #46956), a 4,7 Kb vector expressing the reporter gene EGFP. Voltage and duration of pulses were varied according to the conditions listed in Additional file 1: Table S2. Transfection efficiencies were calculated using the cell image analysis software CellProfiler [75] and are listed in Additional file 1: Table S3. For RNP transfection, RTgutGC cells were seeded at a density of 2 × 10^4^ cells/cm^2^ in 150 cm^2^ flasks (TPP, Transadingen, Switzerland) and incubated for 3 days at 19 ± 1 °C. Next, cells were harvested by trypsinization and washed twice with 5 ml of Opti-MEM™ (Invitrogen, Basel, Switzerland) in order to remove any residual serum. RTgutGC cells were then resuspended in 300 µl of Opti-MEM™ and mixed with the RNP complexes and electroporation enhancer prepared as described in the previous section. Finally, 100 µl of the cells-RNPs mixture was dispensed in three cuvettes with 2 mm gap (EC-002S, Sonidel limited, Ireland). A background, a negative and a positive control were prepared for each electroporation experiment. More specifically, in order to take into account any possible unspecific ATTO™ 550 signal resulting from the unspecific binding of the RNP-fluorophore complex to the cell surface, a background control was included by adding the same amount of RNP complexes to untransfected RTgutGC cells. A negative control, represented by untransfected cells, and a positive control represented by RTgutGC cells electroporated with mRFP-C1 (Addgene plasmid #54,764), were added, respectively. The electroporation system was set with the following parameters: (i) poring pulse: 150 V, 5 ms pulse length, 50 ms pulse interval, 2 pulses, 20% D. rate, + polarity; (ii) transfer pulse: 20 V, 50 ms pulse lenght, 50 ms pulse interval, 5 pulses, 40% D. rate, ± polarity. Immediately after electroporation, 200 µl of L-15 medium supplemented with 10% FCS were added to the electroporation cuvette. The cell suspension was then transferred into a 12 well plate (Greiner Bio-One, Kremsmünster, Austria) containing 500 µl of L-15 medium supplemented with 10% FCS and incubated for 48 h at the conditions described for cell culture above for recovery.

### Fluorescence-activated cell sorting

Following 48 h of recovery, transfected cells were enriched via FACS using ATTO™ 550 signal. More specifically, cells were washed twice with 1 ml of Versene and then detached with 150 µl of trypsin. Following 3 min of incubation, trypsin was inhibited by adding 1 ml of complete L-15 medium. Cells were collected in a tube, centrifuged at 500*g* for 5 min and washed twice with 1 ml of Versene. The cell suspension was finally resuspended in 300 µl of L-15 complete medium and filtered through a 35 μm cell strainer cap (Falcon, USA) in order to remove cell clumps. FACS was performed on BD FACSAria III (BD) using 130 μm nozzle. Sorted cells were recovered in a 96 well plate (Greiner Bio-One, Kremsmünster, Austria) containing 200 µl of L-15 medium supplemented with 10% FCS and conditioned medium in order to facilitate RTgutGC cell attachment and survival following FACS. Conditioned medium was prepared on the day of sorting by harvesting spent complete medium from RTgutGC cells grown for 3 days. The medium was filtered with 0.2-micron syringe filter (Minisart-Plus, Sigma Aldrich, Germany) in order to remove dead cells or debris and added to the 96 well plate at a final concentration of 5%. Sorted cells were incubated at the conditions described for cell culture for 3 weeks and then passaged gradually into plates with larger well sizes.

### T7 Endonuclease I (T7E1) assay

In order to evaluate the overall gene editing efficiency, a T7 endonuclease (T7E1) assay was performed. Genomic DNA was extracted from transfected and untransfected RTgutGC cells using DirectPCR® Lysis Reagent-cell (Viagen Biotech, California, USA) following manufacturer’s instructions. The *cyp1a1* region targeted by the crRNA was amplified using GoTaq DNA polymerase (Promega AG, Switzerland) with primers CYP1A1_EF/CYP1A1_ER (Additional file 1: Table S4), yielding a DNA fragment of 1086 bp. The PCR product was purified using the MinElute PCR Purification Kit (Qiagen, Hilden, Germany) and quantified using a Nanodrop Spectrophotometer (NanoDrop®, ND-1000, ThermoFischer, USA).

Because of the similarity between the selected crRNA and a region of the *cyp1a3* coding gene, T7E1 assay was performed also on this gene. More specifically, amplification of *cyp1a3* gene from untransfected and transfected cells was performed using primers CYP1A3_EF/CYP1A3_ER, yielding a PCR product of 892 bp. Purification and quantification of the PCR product was performed as described above for *cyp1a1*. Finally, as positive control for the T7E1 assay, a mixed PCR amplicon obtained by combining a wild type template with the equivalent amplicon from a known mutant template was included. For the T7E1 assay, 200 ng of PCR product was first heated, then cooled down to room temperature to allow heteroduplex formation as follows: 95 °C for 5 min, from 95° to 85 °C at − 2 °C per second, from 85° to 25 °C at − 0.1 °C per second. Then, 10 units of T7E1 enzyme (New England Biolabs, Massachusetts, USA) were added and the sample incubated at 37 °C for 15 min. At the end of the incubation period, the enzymatic reaction was stopped by adding 1.5 µl of 0.25 M EDTA. Products from the mismatch assay were examined using the 2100 BioAnalyzer on a High Sensitivity DNA Chip (Agilent Technologies, California, USA). Gel preparation and fragments analyses were performed following manufacturer’s instructions. The estimated percentage of gene editing was calculated according to the following formula:

$$\% gene \; editing \; efficiency \;= \;100 \times (1-\sqrt{1-fraction \; cleaved} )$$

More specifically the fraction cleaved was defined as (concentration of digested products) / (concentration of digested products + undigested parental band).

### Sequencing of *cyp1a1* gene and Interference of CRISPR Edits analysis

The *cyp1a1* region targeted by the crRNA was amplified through PCR and purified as described above. Wild type and transfected *cyp1a1* PCR products were Sanger sequenced (Microsynth, Balgach, Switzerland) using primers CYP1A1_EF/CYP1A1_ER. In order to assess the nature and frequency of the CRISPR-mediated edits, Sanger sequencing data were analyzed using the online tool ICE (Synthego, ICE Analysis; https://ice.synthego.com).

### Clonal cell isolation of RTgutGC cells and Sanger sequencing

In order to clonally isolate *cyp1a1* mutant cell lines, transfected RTgutGC cells were seeded at a low density of 600–1200 cells/well in a 6-well plate (Greiner Bio-One, Kremsmünster, Austria) with L-15 medium supplemented with 10% FCS. Following incubation under regular cell culture conditions for one week, each well was examined for the presence of healthy and isolated colonies. The plate was incubated for approximately 3 weeks with a medium change once a week. Small or branched colonies with irregular shapes were avoided as they might not be clonal or have growth defects. Once the colony of interest reached the size of approximately 300–400 cells, it was isolated as follows. The growth medium from each well was discarded and cells washed twice with 1 ml of Versene. Bel-Art™ Cloning Cylinders (Fisher Scientific, Hampton, USA) with a diameter of 4.7 mm were picked using sterile forceps and gently pressed in autoclave-sterile Dow Corning®976 V silicone high vacuum grease (Corning, New York, USA). The cylinder was gently placed over the colony of interest and pressed using the forceps in order to create a seal between the plate and the cylinder. About 10 μl of trypsin were added to the cylinder and incubated for a maximum of 3 min. Following incubation, a few drops of complete growth medium were added to the cylinder in order to stop the enzymatic digestion. The cell suspension was gently aspirated and transferred in a well of a 96-well plate (Greiner Bio-One, Kremsmünster, Austria) containing L-15 medium supplemented with 10% FCS. The cylinder was rinsed with additional medium in order to collect any remaining cells. The 96-well plate was incubated at regular culture conditions and half the medium changed once a week. Cells were gradually sub-cultured into bigger well plates until reaching the 6-well plate stage. Genomic DNA extraction and subsequent amplification of the *cyp1a1* gene via PCR was performed for each isolated colony as previously described. Detection of mutation in the *cyp1a1* gene was performed via PCR amplification using primers CYP1A1_EF/CYP1A1_ER and Sanger sequencing. For each mutant clone, the DNA region of *cyp1a3* identified as potential off-target site was amplified through PCR using primers CYP1A3_EF/CYP1A3_ER (Additional file 1: Table S4) and submitted to Sanger sequencing.

### Assessment of the clonality of the *cyp1a1* mutant cell lines

Once rainbow trout *cyp1a1* mutant clones were identified, their clonality was investigated. The *cyp1a1* region surrounding the CRISPR-targeted area was amplified from the identified gene edited cell lines using primers CYP1A1EF_SmaI/ CYP1A1ER_SmaI (Additional file 1: Table S4), containing engineered SmaI restriction sites. PCR products were purified using MinElute PCR Purification Kit (Qiagen, Hilden, Germany) and quantified using the Nanodrop Spectrophotometer (NanoDrop®, ND-1000, ThermoFischer, USA). The purified *cyp1a1* PCR product from each mutant cell line and the cloning vector pBluescript KS + were digested with SmaI (New England Biolabs, Ipswich, MA, USA). Following linearization, pBluescript KS + was treated with Alkaline Phosphatase (CIP) (New England Biolabs, Ipswich, MA, USA) in order to prevent its re-ligation. Approximately 50 ng of vector were ligated overnight with *cyp1a1* digested products using 40 units of T4 ligase (New England Biolabs, Ipswich, MA, USA). A final molar ratio of 1:6 (plasmid:insert) was used for the ligation reaction. Half of the ligation reaction was transformed into NEB 5-alpha Competent *Escherichia coli* (High Efficiency) (New England Biolabs, Ipswich, MA, USA) following manufacturer’s instructions. Transformed *E. coli* suspension was seeded in Luria–Bertani (LB) (Sigma Aldrich, Germany) plates supplemented with 100 µg/ml of Ampicillin. Ampicillin resistant colonies were screened via colony PCR using KS/SK primers (Additional file 1: Table S4) annealing in the multiple cloning site of pBluescript and able to amplify a fragment of 1125 bp only if the *cyp1a1* insert was correctly cloned. For the colony PCR, *E. coli* colonies were passaged in new LB-agar plates supplemented with ampicillin and then resuspended in 10 µl of H_2_0. A 1 µl aliquot of the cell suspension was added to the PCR reaction. PCR products were run on a 1% agarose gel in Tris Acetate EDTA buffer (TAE), allowing the identification of positive *E. coli* clones. Plasmid DNA was extracted from the positive colonies using PureYield™ Plasmid Miniprep System (Promega AG, Switzerland) and Sanger sequenced (Microsynth, Balgach, Switzerland) using primers KS/SK.

## Supplementary Information

**Additional file 1.** Additional tables and figures.

## Data Availability

The datasets generated and/or analyzed during the current study are available in the online repository, https://doi.org/10.25678/0002YM.

## Abbreviations

CRISPR/Cas9: Clustered Regularly Interspaced Short Palindromic Repeats/CRISPR-associated protein 9
ZFN: Zinc finger nucleases
TALENS: Transcription activator-like effector nucleases
gRNA: Guide RNA
crRNA: Crispr RNA
tracrRNA: Transactivating RNA
PAM: Protospacer adjacent motif
RNP: Ribonucleoprotein
DSB: Double strand break
NHEJ: Non-homologous end joining
FACS: Fluorescence-activated cell sorting
EGFP: Enhanced green fluorescent protein

## References

[1] van Soolingen D, de Haas PE, Hermans PW, Groenen PM, van Embden JD (1993). Comparison of various repetitive DNA elements as genetic markers for strain differentiation and epidemiology of *Mycobacterium tuberculosis*. J Clin Microbiol.

[2] Makarova KS, Grishin NV, Shabalina SA, Wolf YI, Koonin EV (2006). A putative RNA-interference-based immune system in prokaryotes: computational analysis of the predicted enzymatic machinery, functional analogies with eukaryotic RNAi, and hypothetical mechanisms of action. Biol Direct.

[3] Barrangou R, Fremaux C, Deveau H, Richards M, Boyaval P, Moineau S, Romero DA, Horvath P (2007). CRISPR provides acquired resistance against viruses in prokaryotes. Science.

[4] Cong L, Ran FA, Cox D, Lin S, Barretto R, Habib N, Hsu PD, Wu X, Jiang W, Marraffini LA (2013). Multiplex genome engineering using CRISPR/Cas systems. Science.

[5] Jinek M, East A, Cheng A, Lin S, Ma E, Doudna J (2013). RNA-programmed genome editing in human cells. Elife.

[6] Mali P, Yang L, Esvelt KM, Aach J, Guell M, DiCarlo JE, Norville JE, Church GM (2013). RNA-guided human genome engineering via Cas9. Science.

[7] Gaj T, Gersbach CA, Barbas CF (2013). ZFN, TALEN, and CRISPR/Cas-based methods for genome engineering. Trends Biotechnol.

[8] Hefferin ML, Tomkinson AE (2005). Mechanism of DNA double-strand break repair by non-homologous end joining. DNA Repair (Amst).

[9] Capecchi MR (1989). Altering the genome by homologous recombination. Science.

[10] Liu M, Rehman S, Tang X, Gu K, Fan Q, Chen D, Ma W (2018). Methodologies for Improving HDR Efficiency. Front Genet.

[11] Cleveland BM, Yamaguchi G, Radler LM, Shimizu M (2018). Editing the duplicated insulin-like growth factor binding protein-2b gene in rainbow trout (Oncorhynchus mykiss). Sci Rep.

[12] Edvardsen RB, Leininger S, Kleppe L, Skaftnesmo KO, Wargelius A (2014). Targeted mutagenesis in Atlantic salmon (*Salmo salar* L.) using the CRISPR/Cas9 system induces complete knockout individuals in the F0 generation. PLoS ONE.

[13] Wargelius A, Leininger S, Skaftnesmo KO, Kleppe L, Andersson E, Taranger GL, Schulz RW, Edvardsen RB (2016). Dnd knockout ablates germ cells and demonstrates germ cell independent sex differentiation in Atlantic salmon. Sci Rep.

[14] Datsomor AK, Zic N, Li K, Olsen RE, Jin Y, Vik JO, Edvardsen RB, Grammes F, Wargelius A, Winge P (2019). CRISPR/Cas9-mediated ablation of elovl2 in Atlantic salmon (*Salmo salar* L.) inhibits elongation of polyunsaturated fatty acids and induces Srebp-1 and target genes. Sci Rep.

[15] Datsomor AK, Olsen RE, Zic N, Madaro A, Bones AM, Edvardsen RB, Wargelius A, Winge P (2019). CRISPR/Cas9-mediated editing of Delta5 and Delta6 desaturases impairs Delta8-desaturation and docosahexaenoic acid synthesis in Atlantic salmon (*Salmo salar* L.). Sci Rep.

[16] Khalil K, Elayat M, Khalifa E, Daghash S, Elaswad A, Miller M, Abdelrahman H, Ye Z, Odin R, Drescher D (2017). Generation of Myostatin gene-edited channel Catfish (*Ictalurus punctatus*) via zygote injection of CRISPR/Cas9 system. Sci Rep.

[17] Elaswad A, Khalil K, Ye Z, Liu Z, Liu S, Peatman E, Odin R, Vo K, Drescher D, Gosh K (2018). Effects of CRISPR/Cas9 dosage on TICAM1 and RBL gene mutation rate, embryonic development, hatchability and fry survival in channel catfish. Sci Rep.

[18] Li M, Feng R, Ma H, Dong R, Liu Z, Jiang W, Tao W, Wang D (2016). Retinoic acid triggers meiosis initiation via stra8-dependent pathway in Southern catfish *Silurus meridionalis*. Gen Comp Endocrinol.

[19] Chakrapani V, Patra SK, Panda RP, Rasal KD, Jayasankar P, Barman HK (2016). Establishing targeted carp TLR22 gene disruption via homologous recombination using CRISPR/Cas9. Dev Comp Immunol.

[20] Zhong Z, Niu P, Wang M, Huang G, Xu S, Sun Y, Xu X, Hou Y, Sun X, Yan Y (2016). Targeted disruption of sp7 and myostatin with CRISPR-Cas9 results in severe bone defects and more muscular cells in common carp. Sci Rep.

[21] Gratacap RL, Wargelius A, Edvardsen RB, Houston RD (2019). Potential of genome editing to improve aquaculture breeding and production. Trends Genet.

[22] Gratacap RL, Jin YH, Mantsopoulou M, Houston RD (2020). Efficient genome editing in multiple salmonid cell lines using ribonucleoprotein complexes. Mar Biotechnol (NY).

[23] Collet B, Collins C, Lester K (2018). Engineered cell lines for fish health research. Dev Comp Immunol.

[24] Lopez A, Fernandez-Alonso M, Rocha A, Estepa A, Coll JM (2001). Transfection of epithelioma papulosum cyprini (EPC) carp cells. Biotech Lett.

[25] Dehler CE, Boudinot P, Martin SA, Collet B (2016). Development of an efficient genome editing method by CRISPR/Cas9 in a fish cell line. Mar Biotechnol (NY).

[26] Dehler CE, Lester K, Della Pelle G, Jouneau L, Houel A, Collins C, Dovgan T, Machat R, Zou J, Boudinot P (2019). Viral resistance and IFN Signaling in STAT2 knockout fish cells. J Immunol.

[27] Escobar-Aguirre S, Arancibia D, Escorza A, Bravo C, Andres ME, Zamorano P, Martinez V (2019). Development of a bicistronic vector for the expression of a CRISPR/Cas9-mCherry system in fish cell lines. Cells.

[28] Zhao Y, Wang T, Yu Z, Wang H, Liu B, Wu C, Teng CB (2016). Inhibiting cyprinid herpesvirus-3 replication with CRISPR/Cas9. Biotechnol Lett.

[29] Ma J, Fan Y, Zhou Y, Liu W, Jiang N, Zhang J, Zeng L (2018). Efficient resistance to grass carp reovirus infection in JAM-A knockout cells using CRISPR/Cas9. Fish Shellfish Immunol.

[30] Kim MS, Shin MJ, Kim KH (2018). Increase of viral hemorrhagic septicemia virus growth by knockout of IRF9 gene in *Epithelioma papulosum* cyprini cells. Fish Shellfish Immunol.

[31] Xu CL, Ruan MZC, Mahajan VB, Tsang SH (2019). Viral delivery systems for CRISPR. Viruses.

[32] Gratacap RL, Regan T, Dehler CE, Martin SAM, Boudinot P, Collet B, Houston RD (2020). Efficient CRISPR/Cas9 genome editing in a salmonid fish cell line using a lentivirus delivery system. BMC Biotechnol.

[33] David RM, Doherty AT (2017). Viral vectors: the road to reducing genotoxicity. Toxicol Sci.

[34] Kim S, Kim D, Cho SW, Kim J, Kim JS (2014). Highly efficient RNA-guided genome editing in human cells via delivery of purified Cas9 ribonucleoproteins. Genome Res.

[35] Liang X, Potter J, Kumar S, Zou Y, Quintanilla R, Sridharan M, Carte J, Chen W, Roark N, Ranganathan S (2015). Rapid and highly efficient mammalian cell engineering via Cas9 protein transfection. J Biotechnol.

[36] Liang Z, Chen K, Li T, Zhang Y, Wang Y, Zhao Q, Liu J, Zhang H, Liu C, Ran Y (2017). Efficient DNA-free genome editing of bread wheat using CRISPR/Cas9 ribonucleoprotein complexes. Nat Commun.

[37] Liu Q, Yuan Y, Zhu F, Hong Y, Ge R (2018). Efficient genome editing using CRISPR/Cas9 ribonucleoprotein approach in cultured Medaka fish cells. Biol Open.

[38] Fuller SA, Takahashi M, Hurrell JG (2001). Cloning of hybridoma cell lines by limiting dilution. Curr Protoc Mol Biol.

[39] Gross A, Schoendube J, Zimmermann S, Steeb M, Zengerle R, Koltay P (2015). Technologies for Single-Cell Isolation. Int J Mol Sci.

[40] Datta S, Malhotra L, Dickerson R, Chaffee S, Sen CK, Roy S (2015). Laser capture microdissection: big data from small samples. Histol Histopathol.

[41] Brouzes E, Medkova M, Savenelli N, Marran D, Twardowski M, Hutchison JB, Rothberg JM, Link DR, Perrimon N, Samuels ML (2009). Droplet microfluidic technology for single-cell high-throughput screening. P Natl Acad Sci USA.

[42] Edd JF, Di Carlo D, Humphry KJ, Koster S, Irimia D, Weitz DA, Toner M (2008). Controlled encapsulation of single-cells into monodisperse picolitre drops. Lab Chip.

[43] Miltenyi S, Muller W, Weichel W, Radbruch A (1990). High gradient magnetic cell separation with MACS. Cytometry.

[44] Martin BM (1994). Tissue culture techniques: an introduction.

[45] Freshney RI (2005). Cloning and selection. Cult Anim Cells..

[46] McFarland DC (2000). Preparation of pure cell cultures by cloning. Methods Cell Sci.

[47] Mathupala S, Sloan AA (2009). An agarose-based cloning-ring anchoring method for isolation of viable cell clones. Biotechniques.

[48] Lin W, Xu L, Li G (2019). A novel protocol for isolation and culture of multipotent progenitor cells from human urine. J Orthop Translat.

[49] Giuliano CJ, Lin A, Girish V, Sheltzer JM (2019). Generating Single Cell-Derived Knockout Clones in Mammalian Cells with CRISPR/Cas9. Curr Protoc Mol Biol..

[50] Castro R, Martin SA, Zou J, Secombes CJ (2010). Establishment of an IFN-gamma specific reporter cell line in fish. Fish Shellfish Immunol.

[51] Kawano A, Haiduk C, Schirmer K, Hanner R, Lee LEJ, Dixon B, Bols NC (2011). Development of a rainbow trout intestinal epithelial cell line and its response to lipopolysaccharide. Aquacult Nutr.

[52] Schug H, Maner J, Begnaud F, Berthaud F, Gimeno S, Schirmer K, Zupanic A (2019). Intestinal fish cell barrier model to assess transfer of organic chemicals in vitro: an experimental and computational study. Environ Sci Technol.

[53] Schug H, Maner J, Hulskamp M, Begnaud F, Debonneville C, Berthaud F, Gimeno S, Schirmer K (2020). Extending the concept of predicting fish acute toxicity in vitro to the intestinal cell line RTgutGC. Altex.

[54] Wang J, Lei P, Gamil AAA, Lagos L, Yue Y, Schirmer K, Mydland LT, Overland M, Krogdahl A, Kortner TM (2019). Rainbow trout (Oncorhynchus Mykiss) intestinal epithelial cells as a model for studying gut immune function and effects of functional feed ingredients. Front Immunol.

[55] Drieschner C, Konemann S, Renaud P, Schirmer K (2019). Fish-gut-on-chip: development of a microfluidic bioreactor to study the role of the fish intestine in vitro. Lab Chip.

[56] Labun K, Montague TG, Krause M, Torres Cleuren YN, Tjeldnes H, Valen E (2019). CHOPCHOP v3: expanding the CRISPR web toolbox beyond genome editing. Nucleic Acids Res.

[57] Jinek M, Chylinski K, Fonfara I, Hauer M, Doudna JA, Charpentier E (2012). A programmable dual-RNA-guided DNA endonuclease in adaptive bacterial immunity. Science.

[58] Shapiro J, Iancu O, Jacobi AM, McNeill MS, Turk R, Rettig GR, Amit I, Tovin-Recht A, Yakhini Z, Behlke MA (2020). Increasing CRISPR efficiency and measuring its specificity in HSPCs using a clinically relevant system. Mol Ther Methods Clin Dev.

[59] Wu X, Kriz AJ, Sharp PA (2014). Target specificity of the CRISPR-Cas9 system. Quant Biol.

[60] Jeggo PA (1998). DNA breakage and repair. Adv Genet.

[61] Lieber MR (2010). The mechanism of double-strand DNA break repair by the nonhomologous DNA end-joining pathway. Annu Rev Biochem.

[62] Brinkman EK, Chen T, Amendola M, Steensel B (2014). Easy quantitative assessment of genome editing by sequence trace decomposition. Nucleic Acids Res.

[63] Brinkman EK, van Steensel B (2019). Rapid quantitative evaluation of CRISPR genome editing by TIDE and TIDER. Methods Mol Biol.

[64] Synthego Performance Analysis, ICE Analysis. 2019. v2.0. Synthego.

[65] Grainger S, Lonquich B, Oon CH, Nguyen N, Willert K, Traver D (2017). CRISPR guide RNA validation in vitro. Zebrafish.

[66] Horlbeck MA, Witkowsky LB, Guglielmi B, Replogle JM, Gilbert LA, Villalta JE, Torigoe SE, Tjian R, Weissman JS (2016). Nucleosomes impede Cas9 access to DNA in vivo and in vitro. Elife.

[67] Chen X, Rinsma M, Janssen JM, Liu J, Maggio I, Goncalves MA (2016). Probing the impact of chromatin conformation on genome editing tools. Nucleic Acids Res.

[68] Pace JK, Sen SK, Batzer MA, Feschotte C (2009). Repair-mediated duplication by capture of proximal chromosomal DNA has shaped vertebrate genome evolution. PLoS Genet.

[69] Ricchetti M, Fairhead C, Dujon B (1999). Mitochondrial DNA repairs double-strand breaks in yeast chromosomes. Nature.

[70] D'Anjou H, Chabot C, Chartrand P (2004). Preferential accessibility to specific genomic loci for the repair of double-strand breaks in human cells. Nucleic Acids Res.

[71] Lujan H, Romer E, Salisbury R, Hussain S, Sayes C (2020). Determining the biological mechanisms of action for environmental exposures: applying CRISPR/Cas9 to toxicological assessments. Toxicol Sci.

[72] Pickar-Oliver A, Gersbach CA (2019). The next generation of CRISPR-Cas technologies and applications. Nat Rev Mol Cell Biol.

[73] Wang T, Wei JJ, Sabatini DM, Lander ES (2014). Genetic screens in human cells using the CRISPR-Cas9 system. Science.

[74] Tsai SQ, Zheng Z, Nguyen NT, Liebers M, Topkar VV, Thapar V, Wyvekens N, Khayter C, Iafrate AJ, Le LP (2015). GUIDE-seq enables genome-wide profiling of off-target cleavage by CRISPR-Cas nucleases. Nat Biotechnol.

[75] Carpenter AE, Jones TR, Lamprecht MR, Clarke C, Kang IH, Friman O, Guertin DA, Chang JH, Lindquist RA, Moffat J (2006). Cell Profiler: image analysis software for identifying and quantifying cell phenotypes. Genome Biol.

