# Supplementary material for: A ribonucleoprotein transfection strategy for CRISPR/Cas9‐mediated gene editing and single cell cloning in rainbow trout cells
Source: Cell Biosci. 2021 Jun 3;11:103. doi: 10.1186/s13578-021-00618-0 (PMC8176604; doi:10.1186/s13578-021-00618-0)
Supplement: Supplementary file 1 — Additional file 1. Additional tables and figures. [file 13578_2021_618_MOESM1_ESM.docx]

**A ribonucleoprotein transfection strategy for CRISPR/Cas9‐mediated gene editing and single cell cloning in rainbow trout cells**

Marina Zoppo^1*^, Nicole Okoniewski^1^, Stanislav Pantelyushin^2^, Johannes vom Berg^2^, Kristin Schirmer^1,3,4^

^1^Eawag, Swiss Federal Institute of Aquatic Science and Technology, 8600 Dübendorf, Switzerland

^2^Institute of Laboratory Animal Science, University of Zurich, 8952 Schlieren, Switzerland

^3^EPF Lausanne, ENAC, 1015 Lausanne, Switzerland

^4^ETH Zürich, Department of Environmental Systems Science, 8092 Zürich, Switzerland

*: corresponding author [marina.zoppo@eawag.ch](mailto:marina.zoppo@eawag.ch)

**SUMMARY OF SUPPLEMENTARY INFORMATION**

**Supplementary Figures**

Fig.S1: RTgutGC cells FACS results…………………………………………………………………………………………………………………………….2

Fig. S2: Titration experiment of RTgutGC cells sorted via FACS…………………………………………………………………………………3

**Supplementary Tables**

Table S1: Predicted guide sequences for *cyp1a1…*……………………………………………………………………………………………….….4-5

Table S2: Optimization of the electroporation parameters for RTgutGC transfection……………………………………….………..6

Table S3: Transfection efficiencies obtained in the optimization experiment……………………………………………………..………7

Table S4: List of the primers used in this study……………………………………………………………………………………………………………8


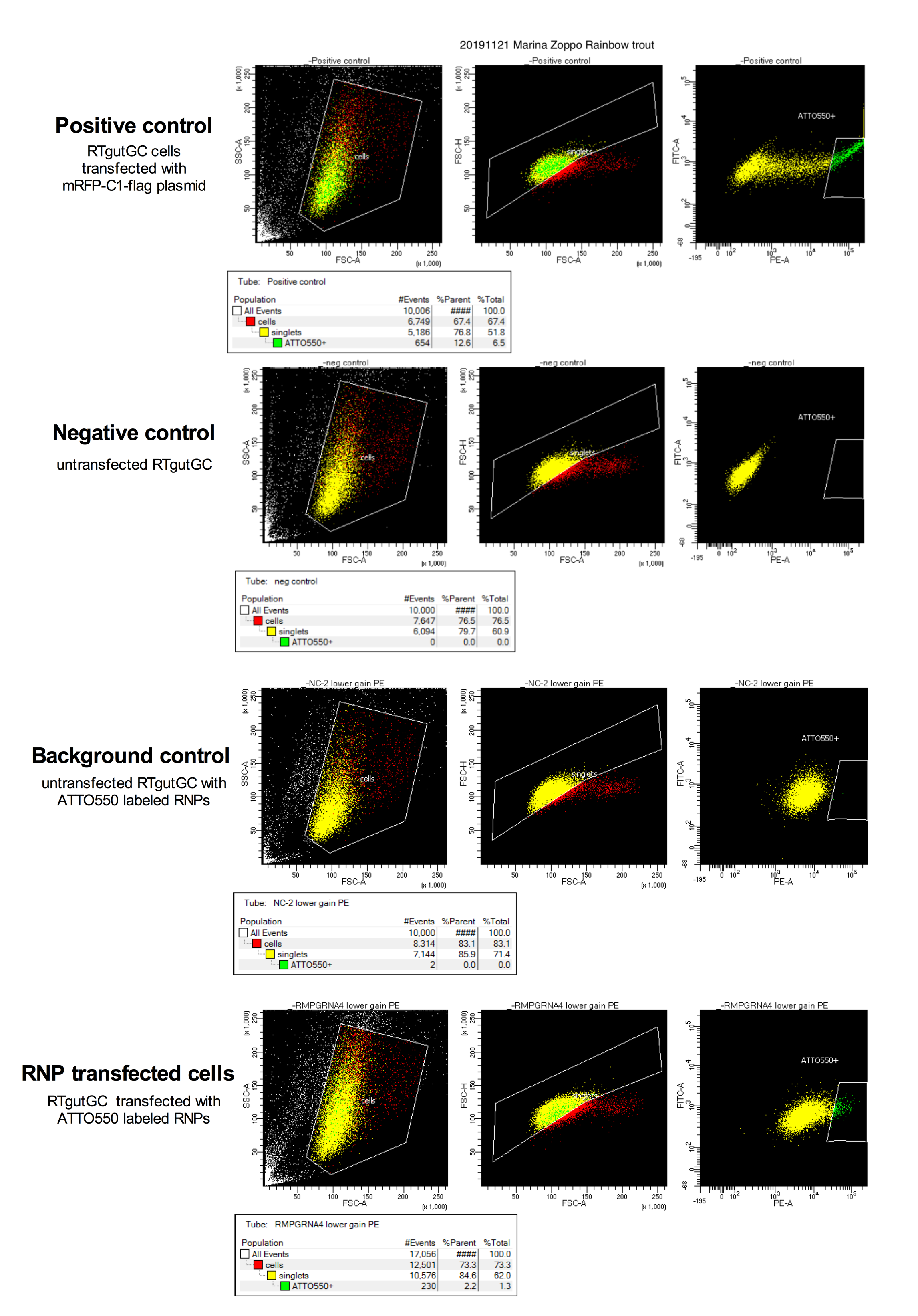


**Fig. S1: RTgutGC cells FACS results.** In order from the top, positive control (cells transfected with mRFP-C1-flag plasmid), negative control (untransfected cells), background control (untransfected cells and RNPs) and transfected cells. Each dot in the plots represents an event (cell) with respect to the x and y axis of the plot. For each panel, starting from the left: (i) Forward versus (FCS-A) vs side scatter (SSC-A) plot depicting the distribution of cells based upon size and complexity; (ii) forward scatter height (FSC-H) vs. forward scatter area (FSC-A) density plot indicating singlets and doublets cells; (iii) FITC-A vs PE-A which indicates the ATTO™ 550 fluorescence of the cells.


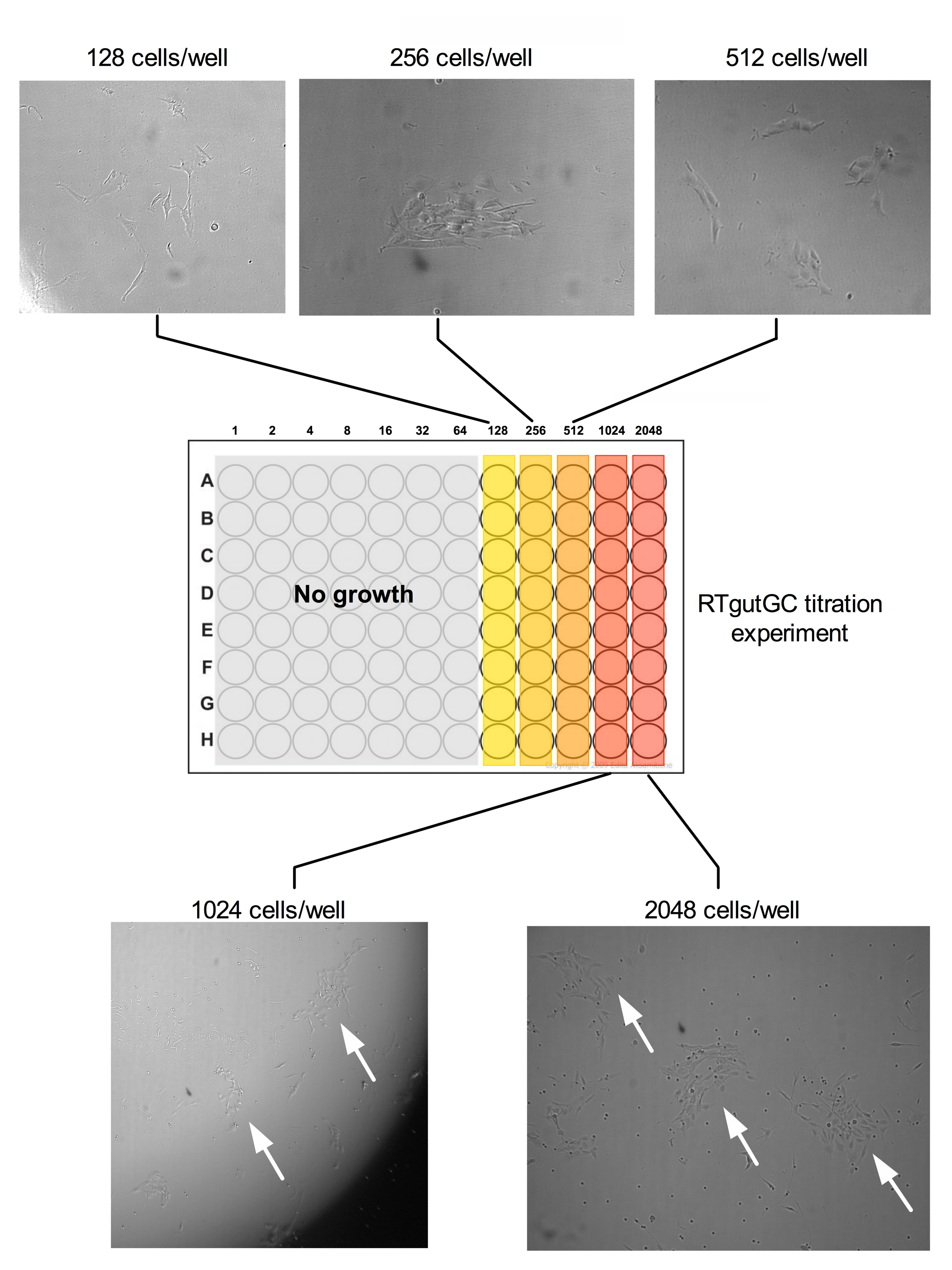


**Fig. S2: Titration experiment of RTgutGC cells sorted via FACS.** RTgutGC cells were sorted in a 96 well plate at increasing densities (from 1 cell/well to 2048 cells/well), including 6 replicates/density. After one week of incubation in L-15 medium supplemented with 10% FCS and 5% conditioned medium, no viable cells were detected when less than 128 cells were sorted in the well (grey zone of the 96 well plate). On the contrary, increasing cell attachment and colony formation were observed in the wells with 128, 256, 512, 1024 and 2048 cells/well. Micrographs were taken at the inverted microscope.

**Table S1: Predicted guide sequences for *cyp1a1.***

| **Rank** | **Target sequence** | **Genomic location** | **Strand** | **GC content (%)** | **Self-complem.** | **Off-targets*** | | | | **Efficiency** |  |
| --- | --- | --- | --- | --- | --- | --- | --- | --- | --- | --- | --- |
|  |  |  |  |  |  | **MM0** | **MM1** | **MM2** | **MM3** |  | **Comments** |
| 1 | GCCACTGACATCCATGACGGAGG | NC_035082.1:69563571 | - | 60 | 0 | 0 | 0 | 0 | 1 | 70.87 | Off-target in the gene LOC110534857 encoding for the BRCA2-interacting transcriptional repressor EMSY-like. |
| 2 | GAAGACCTCGTTGCGTCCGATGG | NC_035082.1:69565122 | - | 60 | 2 | 0 | 0 | 1 | 0 | 59.06 | Off-target in the *cyp1a3* gene. Moreover, the on target region is located in the last exon of *cyp1a1.* |
| **3** | **CCGCCATATTGTCGTATCGGTGG** | **NC_035082.1:69563608** | **+** | **55** | **0** | **0** | **1** | **0** | **0** | **61.61** | **Off-target located in *cyp1a3*. Sequence selected for CRISPR/Cas9 gene editing** |
| 4 | AGGCTAGCATGCGGCCATGGGGG | NC_035082.1:69565257 | + | 65 | 3 | 0 | 1 | 0 | 0 | 62.16 |  |
| 5 | CTTCCGCCATATTGTCGTATCGG | NC_035082.1:69563605 | + | 45 | 0 | 0 | 1 | 0 | 0 | 54.02 |  |
| 6 | CGCTCTTTCGCCACACTGGAGGG | NC_035082.1:69563474 | + | 60 | 0 | 0 | 1 | 0 | 0 | 51.83 |  |
| 7 | TGGCAATGAGACAGTCCGCCAGG | NC_035082.1:69563308 | + | 60 | 0 | 0 | 1 | 0 | 0 | 46.54 |  |
| 8 | ACTGCCATGAGCGAGCGCTACGG | NC_035082.1:69563237 | + | 60 | 1 | 1 | 0 | 0 | 0 | 66.51 |  |
| 9 | AAAGTGCTCGTATTCGGCATGGG | NC_035082.1:69565076 | + | 45 | 0 | 0 | 0 | 1 | 1 | 60.79 |  |
| 10 | GGGCGCTCATAGCTAGCTTGCGG | NC_035082.1:69563449 | - | 60 | 2 | 1 | 0 | 0 | 0 | 62.76 |  |
| 11 | AGCGCTTGTGCTTCATGGTGAGG | NC_035082.1:69565225 | - | 55 | 0 | 1 | 0 | 0 | 0 | 60.45 |  |
| 12 | GAAAGTGCTCGTATTCGGCATGG | NC_035082.1:69565075 | + | 50 | 0 | 0 | 0 | 1 | 1 | 55.35 |  |
| 13 | AAGCAAGGGGAAGACTTCGCCGG | NC_035082.1:69563339 | + | 55 | 2 | 1 | 0 | 0 | 0 | 57.23 |  |
| 14 | TGAGCCGTAGCGCTCGCTCATGG | NC_035082.1:69563241 | - | 65 | 2 | 1 | 0 | 0 | 0 | 57 |  |
| 15 | TGTGGCTTATCCAGAGATCCAGG | NC_035082.1:69564270 | + | 50 | 0 | 0 | 0 | 1 | 1 | 52.77 |  |
| 16 | CGCCAGGCTCTTATCAAGCAAGG | NC_035082.1:69563324 | + | 55 | 0 | 1 | 0 | 0 | 0 | 52.66 |  |
| 17 | AAAATCCTACTCACGGGTCATGG | NC_035082.1:69564836 | - | 45 | 0 | 1 | 0 | 0 | 0 | 52.44 |  |
| 18 | TCCAGCACATTCCCGATGATGGG | NC_035082.1:69563186 | - | 50 | 0 | 1 | 0 | 0 | 0 | 51.97 |  |
| 19 | CGCTCATAGCTAGCTTGCGGCGG | NC_035082.1:69563446 | - | 60 | 2 | 1 | 0 | 0 | 0 | 44.77 |  |
| 20 | ACCTCCGTCATGGATGTCAGTGG | NC_035082.1:69563570 | + | 55 | 0 | 0 | 1 | 0 | 1 | 62.32 |  |
| 21 | GAGGGACTAAAACGGCTCCCAGG | NC_035082.1:69563150 | + | 60 | 4 | 0 | 1 | 0 | 1 | 57.96 |  |
| 22 | CAAGCGCCGCTGCATCGGTGAGG | NC_035082.1:69565099 | + | 70 | 1 | 0 | 1 | 0 | 1 | 51.14 |  |
| 23 | GGAAGCGCAGCCTTTGGAGCAGG | NC_035082.1:69565159 | - | 65 | 0 | 0 | 1 | 0 | 1 | 48.37 |  |
| 24 | TGAAGGCTAGCATGCGGCCATGG | NC_035082.1:69565254 | + | 60 | 2 | 0 | 1 | 0 | 1 | 42.51 |  |
| 25 | GTCAACCATGACCCGTGAGTAGG | NC_035082.1:69564831 | + | 55 | 1 | 1 | 0 | 0 | 1 | 67.51 |  |
| 26 | TGGGGTCGTTCCCTCCAGTGTGG | NC_035082.1:69563484 | - | 65 | 0 | 0 | 0 | 1 | 2 | 66.02 |  |
| 27 | CAGCACTCAGGAAACGGTCAGGG | NC_035082.1:69565021 | - | 55 | 0 | 1 | 0 | 0 | 1 | 63.29 |  |
| 28 | TGCGGTTGGGCAGGTAACGAAGG | NC_035082.1:69563764 | - | 60 | 0 | 1 | 0 | 0 | 1 | 61.03 |  |
| 29 | CCAAATAGGGATGCGGCCTGTGG | NC_035082.1:69563275 | + | 60 | 1 | 1 | 0 | 0 | 1 | 59.51 |  |
| 30 | GCTGTATAGATCGGGCCTCCCGG | NC_035082.1:69563358 | - | 60 | 1 | 1 | 0 | 0 | 1 | 55.11 |  |

*****: mismatch (MM)

**Table S2: Optimization of the electroporation parameters for RTgutGC transfection.**


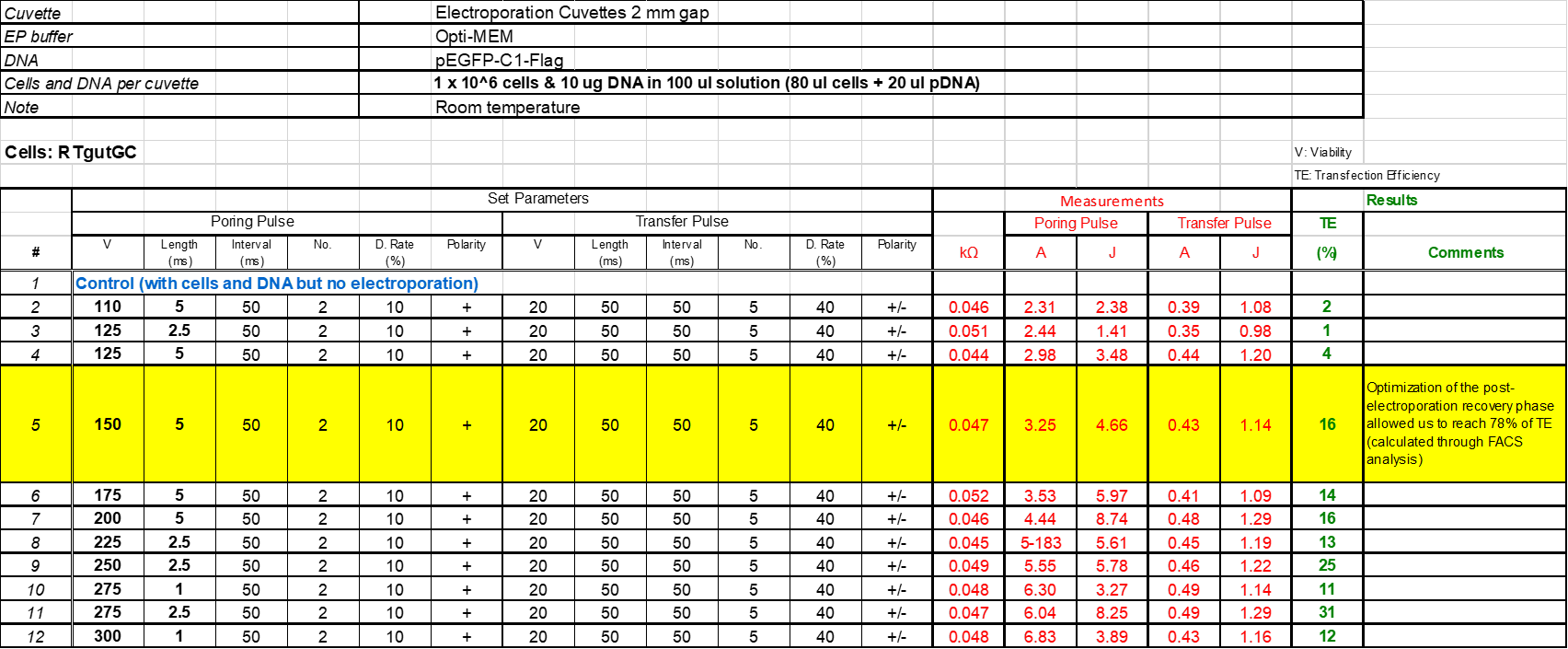


**Table S3: Transfection efficiencies obtained in the optimization experiment.**

**Table S4: List of the primers used in this study.**

| **Primer name** | **Primer sequence (5’🡪 3’)** | **Usage** |
| --- | --- | --- |
| CYP1A1_EF | GGGCTGTATGCGTAAAGCATG | Forward primer for *cyp1a1* amplification and sequencing |
| CYP1A1_ER | CATGGAATGAAACATGTAACA | Reverse primer for *cyp1a1* amplification and sequencing |
| CYP1A3_EF | TGAAACGGCTCCCAGGACC | Forward primer for *cyp1a3* amplification and sequencing |
| CYP1A3_ER | CAGACACCTGGACGTTGGCG | Reverse primer for *cyp1a3* amplification and sequencing |
| CYP1A1_ EF_SmaI | CAGATGAC**CCGCGG**TTATCA* | Cloning of gene-edited *cyp1a1* into pBluescript SK |
| CYP1A1_ ER_SmaI | TCTGGGGTC**CGCGG**TTATCA* | Cloning of gene-edited *cyp1a1* into pBluescript SK |
| KS | CGAGGTCGACGGTATCG | Screening and sequencing of *E. coli* recombinant clones |
| SK | TCTAGAACTAGTGGATC | Screening and sequencing of *E. coli* recombinant clones |

* SmaI restriction sites are highlighted in bold.
